# Diagnosing and treating rheumatic and musculoskeletal diseases in low resource settings: a review of the challenges and opportunities in Tanzania

**DOI:** 10.1093/rap/rkad079

**Published:** 2023-09-23

**Authors:** Francis Furia, Mwidimi Ndosi

**Affiliations:** School of Clinical Medicine, Muhimbili University of Health and Allied Sciences, Dar es salaam, Tanzania; Department of Pediatrics, Muhimbili National Hospital, Dar es Salaam, Tanzania; School of Health and Social Wellbeing, College of Health, Science and Society, University of the West of England, Bristol, UK; Rheumatology Department, University Hospitals Bristol and Weston NHS Foundation Trust, Bristol, UK

Care for people with rheumatic and musculoskeletal diseases (RMDs) has improved in the developed world. However, in many low- and middle-income countries (LMICs), these diseases are underdiagnosed, undertreated and continue to cause disability and cardiovascular-related morbidity and mortality, representing a global health inequality in rheumatology care. We discuss the challenges of diagnosing and treating RMDs in low-resource settings, with Tanzania as an exemplar.

The actual burden of RMDs in LMICs is largely unknown, but few studies suggest a prevalence comparable to that found elsewhere in the world [1–3]. Common RMDs reported among adults in Africa include gout, RA, SLE and SSc [3]. The impact of RMDs on children is underresearched, with prevalent conditions being juvenile arthritis, SLE and dermatomyositis [4].

Most RMDs are closely linked with infectious diseases, which may cause or complicate their presentation, particularly tuberculosis and HIV, and these present difficulties in managing RMDs with immunosuppressive therapies [5]. The complex nature of most RMDs and their presentations may result in affected patients with early symptoms, including pain, stiffness and fatigue, to refrain from seeking care in health facilities. This is more likely in regions with a strong influence of religion and cultural norms in people’s lives. A wide range of beliefs exists about the cause of RMDs, including exposure to cold, old age, demons, witchcraft and sex [6]. In response to an RMD, patients are likely to consult traditional and spiritual healers, and when they decide to seek medical help, they may combine herbal remedies with biomedical treatments.

When patients present at a dispensary or health centre, it is unlikely that the primary healthcare providers (PHPs) will suspect an autoimmune disease, as these symptoms are also present in common endemic infections. Limited knowledge and skills of PHPs and the high burden of infectious diseases presenting with RMD manifestations mean that the diagnosis may be missed or delayed at early stages of a serious RMD. Delayed diagnosis and lack of access to care result in a higher burden of disease and worse outcomes.

Diagnosis of RMDs requires clinical expertise, imaging and inflammatory and serological tests. Tanzania and most LMICs have no formal rheumatology training necessary for the early diagnosis and appropriate management of inflammatory arthritis and CTDs [2, 7]. Apart from X-rays and tests for inflammatory markers, sensitive imaging and serological tests are very expensive and may not be covered by the National Health Insurance Fund. This has implications for confirming diagnosis, especially in early disease.

Management of most RMDs is based on recommendations developed by rheumatology associations in high-income countries, mostly the European Alliance of Associations for Rheumatology, American College of Rherumatology and Paediatric Rheumatology European Society. While these recommendations are evidence-based, they are underpinned by research conducted in high-income countries. Lack of rheumatology research in LMICs and unavailability of interventions included in recommendations limits their applicability, adding to the challenges in clinical management. An interview with physicians in 29 African countries identified difficulties in effective use of methotrexate, such as limited prescribers, inconsistent supply and issues with monitoring, among other challenges [8].

With treatments that take a long time to have beneficial effects, non-adherence adds to the complexity of disease management. In Europe, 30–80% of patients do not adhere to treatment, and the rate is likely higher in LMICs due to inconsistent supplies, cultural beliefs and low levels of health literacy about RMDs. Non-adherence has multiple causes, so culturally and context-appropriate patient education is needed. This requires access to a skilled rheumatology multidisciplinary workforce.

The rheumatology workforce is limited globally, and the situation is particularly worse in LMICs. Tanzania, a country of 60 million people has only one qualified rheumatologist and no rheumatology-trained nurses or allied health professionals. The lack of a trained rheumatology multidisciplinary team makes it difficult to provide evidence-based, holistic and person-centred care.

The challenges of rheumatology care in LMICs provide unique opportunities for research, training of healthcare workers and the development of effective and efficient services.

Research conducted in high-income countries may not translate well in LMICs due to great genetic variability and cultural diversity [9], both of which have implications in disease progression, treatment response and preferred patient outcomes. This presents an opportunity for clinical trials and observational research to stratify patients by risk for specific health outcomes, study response to treatments and develop culturally appropriate outcome measures.

Studying non-pharmacological interventions in these contexts is also necessary, as contextual, cultural and behavioural factors play an important role in disease progression, management and response to treatment. Research would help provide evidence-based and context-appropriate clinical recommendations that are likely to be effective in these populations. Also, research conducted in low-resource contexts will benefit the international recommendations by increasing their external validity.

Implementation studies and service evaluations are also necessary to ensure recommendations and interventions are applicable and working in low-resource settings. Here rheumatology associations can play an important leadership role in advocacy, education and facilitating regional and international collaboration among professional and patient organizations. This will also support the development of evidence-based and culturally appropriate guidelines to address the discrepancy of care in LMICs.

There is a need for reviewing curricula for primary healthcare training, with inclusion of rheumatology skills for early identification of patients with RMDs. Rheumatology specialty training is needed for doctors, nurses, physiotherapists, occupational therapists and radiologists in secondary care to enable them to manage referrals from primary care. Long-term strategies for collaborative training between institutions in high-income countries and LMICs will help address the rheumatology skill shortage [10].

Rheumatology training should be matched with the development of resources to support patients to manage their illness, treatments and lifestyle changes required to maximize their health and well-being. Establishing patient associations will help ensure RMDs are recognized and prioritized by policymakers.

In conclusion, LMICs face a disproportionately high burden of RMDs due to limited resources for diagnosis and treatments. Opportunities for research, training and service improvement are summarised in Table 1. Enhanced collaboration between professional associations, patient associations and institutions across different economic levels will help ensure that all RMDs are recognised, diagnosed and ultimately prevented or cured globally.

**Table 1.** Challenges and opportunities for improving rheumatology care for in LMICs

Challenges	Opportunities	Key stakeholders
Limited rheumatology workforce	Short courses for rheumatology	International associations for rheumatology,^a^ national rheumatology societies, charitable organizations
	Fellowships and internships in well-established rheumatology centres	International associations for rheumatology
	Online rheumatology training	International associations for rheumatology, national rheumatology societies, healthcare professionals, healthcare institutions
	Enhance rheumatology in undergraduate medicine curricula	Medical universities and healthcare training institutions, ministries of health
	Develop curricula for training primary care health workers	Healthcare training institutions, ministries of health
	Advocate for rheumatology as a viable career	National rheumatology societies, healthcare institutions, charitable organisations, rheumatology patient groups
Lack and inconsistent supply of drugs and laboratory facilities for managing RMDs	Registration of drugs for RMDs in LMICs	National drug regulatory authorities, ministries of health, pharmaceutical companies
	Bulk or regional procurement of drugs and laboratory tests	Ministries of health, health facilities
	Include RMD treatments in health insurance policies	Ministries of health
Poor management of RMDs	Development of evidence-based and context-specific treatment guidelines	International Associations for Rheumatology, National Rheumatology Societies, Ministries of health, Healthcare institutions
	Establishment of national rheumatology societies	International Rheumatology Societies, Ministries of Health, Healthcare professionals
	Develop monitoring tools, including patient-reported outcome measures, that are context and culturally appropriate	International associations for rheumatology, national rheumatology societies, ministries of health, healthcare institutions, health facilities
Lack of rheumatology research evidence from LMICs	Conduct research in LMICs to generate evidence that is applicable to this population	Research and healthcare institutions in LMICs in collaboration with those in high-income countries, international associations for rheumatology, national rheumatology societies, charitable organisations, ministries of health, World Health Organization
	Conduct adaptation and implementation studies to ensure recommendations are context and culturally appropriate	Research and healthcare institutions in the LMICs, national rheumatology societies
Misconceptions of RMDs in the society and by policymakers	Establishment of patient associations	Ministries of health, national rheumatology societies
	Establishment of civil societies for advocacy	International rheumatology societies
	Raise awareness and provide health education to the public on the impact of RMDs on individuals, families and society	Ministries of health, medical universities and training institutions, media, health facilities, patient associations
	Highlight the impact of RMDs as a priority for policymakers to prevent disability, morbidity mortality and impact to the economy	National rheumatology societies, patient associations and civil societies, international rheumatology societies

a International associations for rheumatology include but is not limited to the American College of Rheumatology (ACR), African League of Association for Rheumatology (AFLAR), Asia Pacific League of Associations for Rheumatology (APLAR), British Society for Rheumatology (BSR), European Alliance of Associations for Rheumatology (EULAR), International League of Associations for Rheumatology  (ILAR), Paediatric Society of the African League Against Rheumatism (PAFLAR) and Paediatric Rheumatology European Society (PRES).

## Data Availability

No new data were generated or analysed in support of this work.
